# Elbow Evaluation Via Telephone and Video Visit

**DOI:** 10.7759/cureus.39843

**Published:** 2023-06-01

**Authors:** Rock P Vomer, David Carfagno, Adam Lewno, Neil P Shah, Bryan A Farford, Lisa Kieneker, George G. A Pujalte

**Affiliations:** 1 Family Medicine, Mayo Clinic Jacksonville Campus, Jacksonville, USA; 2 Family and Community Health, Orthopedics, Sports Medicine, Duke University, Durham, USA; 3 Sports Medicine, Scottsdale Sports Medicine Institute, Scottsdale, AFG; 4 Sports Medicine, University of Michigan, Ann Arbor, USA; 5 Family and Community Medicine, Mayo Clinic, Jacksonville, USA; 6 Family Medicine, Mayo Clinic, Jacksonville, USA; 7 Family Medicine, Orthopedics, and Sports Medicine, Mayo Clinic, Jacksonville, USA

**Keywords:** elbow, telehealth, functional exam, virtual encounter, orthopedic exam

## Abstract

Background

Elbow conditions and pathology are commonly seen in the outpatient clinic. Telephone and video visits can allow for expeditious assessment of elbow complaints, without the added challenges of commuting for a clinic-based evaluation. In the setting of a pandemic, the benefits of telemedicine are apparent, but the time and effort saved from being able to remotely evaluate musculoskeletal conditions are also useful in a non-pandemic situation. In this modern era of telemedicine, protocols need to be developed to provide guidance for a remote elbow evaluation. As with all musculoskeletal conditions, the history about the elbow complaint allows the clinician to develop a differential diagnosis, which is either supported or refuted based on physical examination and diagnostic studies. Appropriate questions asked over a telephone call can provide answers that lead the clinician to a specific diagnosis and treatment plan. Furthermore, responses to these same questions can be further supported by a video assessment of the affected elbow, which may provide additional evidence to support a diagnosis and plan of care.

Aims

To outline possible questions, responses, and video examination techniques to aid the clinician in elbow examinations conducted via telemedicine.

Methods

We have created a pathway for step-by-step evaluation to help physicians direct their patients through the typical elements of a thorough elbow examination via telehealth.

Results

We have created tables of questions, answers, and instructions to help guide the physician through different aspects of a telehealth elbow examination. We have also included a glossary of descriptive images that demonstrate each maneuver.

Conclusion

This article provides a structured guide to efficiently extracting clinically relevant information during telemedicine examinations of the elbow.

## Introduction

Elbow conditions are commonly seen in primary care clinics and can be concerning in the context of sports and recreational activities [1]. Some conditions in children and adolescents can lead to elbow pain and have dire consequences if not diagnosed and managed early [2]. Sports considerations are important for youth athletes, and the ability to evaluate across a distance is helpful for teams while traveling.

Telephone and video visits allow for expeditious evaluation of elbow complaints [3] without the added difficulty of having to drive or transport a patient already in pain or distress. Evaluating the range of motion (ROM) and determining the mechanism of injury can be done effectively via telemedicine [4]. Appropriate imaging modalities can be ordered based on history and physical examination completed with telemedicine [5]. Telemedicine for elbow complaints is especially appropriate as many times, imaging is not needed for the more common elbow conditions, such as medial and lateral epicondylitis [6]. When imaging is needed, there are specific findings that can be described via telephone or seen over the video, such as decreased ROM or marked swelling, that can assist clinicians in determining the appropriate imaging study to order [7].

In the setting of a pandemic, the utility of telemedicine becomes apparent, given the restriction on travel and requirements for social distancing to prevent the spread of communicable diseases. However, the time and effort saved from being able to remotely evaluate musculoskeletal conditions is also useful in a non-pandemic situation. As noted above, transporting an injured patient can be challenging, and elbow pain typically does not require an ambulance. Parents or friends often need to be available to provide important historical details that aid in making a correct diagnosis. Furthermore, patient with an elbow injury that attempts to drive may put themselves or others at risk due to physical limitations from the injury.

In this modern era of telemedicine, protocols should be developed to provide guidance on how to appropriately evaluate an elbow complaint remotely. These protocols could be beneficial as part of the academic criteria for students training to become medical professionals as telemedicine evolves as another means of health care delivery. Technology will no doubt continue to improve through the years. For example, goniometers are being made available through video [8] or the development of mobile applications that make telemedicine even more accessible to patients [9]. Insurance coverage will likely also be more available, with growing evidence that such visits prevent progression to more serious conditions that will need greater remuneration [10]. In terms of the elbow and upper extremity conditions, telephone and video visits can provide effective triaging and reduce unnecessary procedures, imaging, and referrals. Additionally, these visits provide care that is convenient and more accessible to the patient.

The elbow is a smaller joint, and active ROM testing is revealing and may change management in a meaningful way [11]. ROM is readily visible on video visits, although it may be more challenging to assess during a telephone visit.

## Materials and methods

Evaluating the elbow by telephone

When evaluating the elbow by telephone, there is no physical examination, only self-reported findings and responses to questions posed to patients. Each question should be aimed at specifically uncovering a suspected injury or condition, and each response should be taken in the context of the patient’s history. The responses will require mental visualization on the part of the clinician. This image may be adjusted to fit the picture of the patient formed through their history. For example, if the history and each response appear to be suggesting common extensor tendinopathy, the standardized patient imagined may be adjusted to one with pain over the lateral epicondyle on the affected elbow.

Patient history and physical examination are intimately related, as the history allows the clinician to develop a differential diagnosis that is either supported or refuted based on the physical examination and diagnostic studies. Further questioning prompted by the patient’s response during the physical examination aids in uncovering a suspected injury or condition, creating an accurate differential diagnosis. 

**Figure 1:**
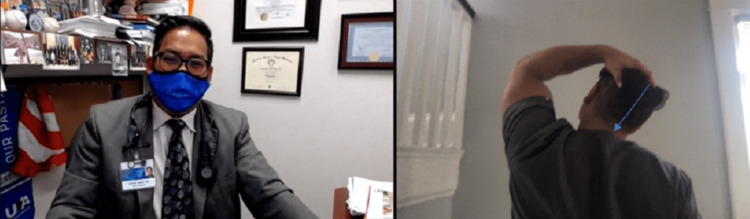
Modified Spurling's Maneuver. Instruct the patient to look over the non-involved shoulder, extend the cervical spine and apply axial compression (arrow) using the non-involved extremity. The reproduction of numbness and/or pain would result in a positive test.

Prior to a focused elbow examination, cervical radiculopathy should be ruled out; this can be done with the Modified Spurling's Test (Figure 1). 

Elbow evaluation by video

**Figure 2:**
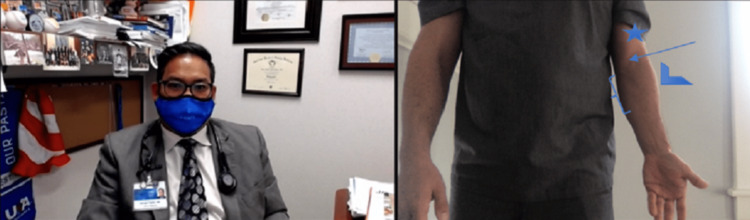
Anterior view of the elbow. Image shows bicep brachii (star) and common bicep tendon (arrow), brachioradialis (arrowhead), and the origin of common flexor tendors (bracket).

**Figure 3:**
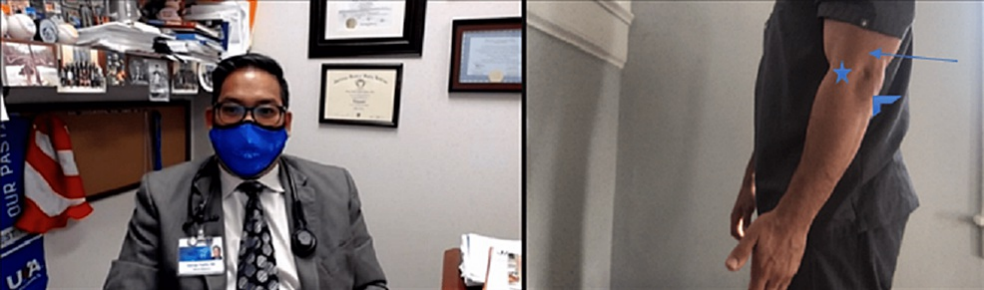
Lateral view of the left elbow. Image shows the triceps (arrow), olecranon (arrowhead), and lateral epicondyle (star).

First, the elbows should be exposed and inspected from the front, side, and back. Any asymmetry should be noted. Malalignment, atrophy, swelling, ecchymosis, and venous distension should be noted, if present (Figures 2, 3).

**Figure 4:**
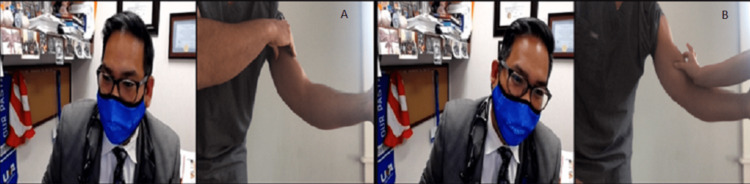
Palpation of bicep. (A) If alone, instruct the patient to use the non-involved extremity to palpate the involved extremity to assess for muscle defects. (B) If the patient is accompanied by another person have that person palpate the involved extremity to assess for muscle defects.

**Figure 5:**
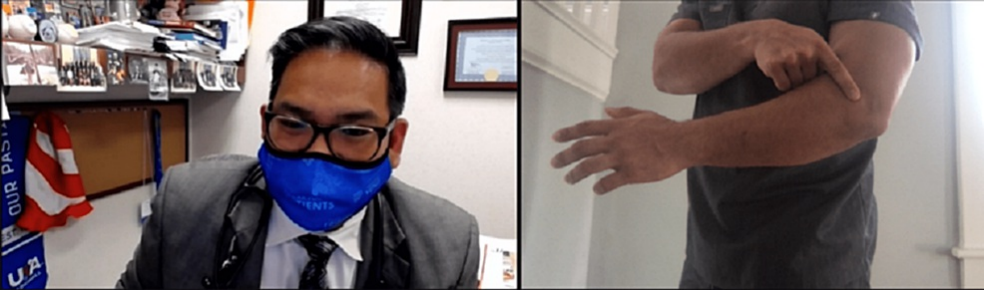
Palpation of the radial head and common extensor origin. If the patient has pain to palpation over the radial head (star) or common extensor origin, this could mean common extensor tendon, radial head, radial collateral ligament, and radial tunnel-nerve pathology.

Palpation cannot be performed by the clinician, but the patient can self-palpate with guidance from the physician. The patient must self-palpate for tenderness over the lateral epicondyle, the radial head, the distal biceps and supinator tendon, the pronator tendon, the common flexor tendon, the ulnar collateral ligament (UCL) location, the medial epicondyle, the medial triceps, the cubital tunnel, the olecranon, the triceps tendon, and 2 to 5 cm distal to the lateral epicondyle (Figures 4, 5).

**Figure 6:**
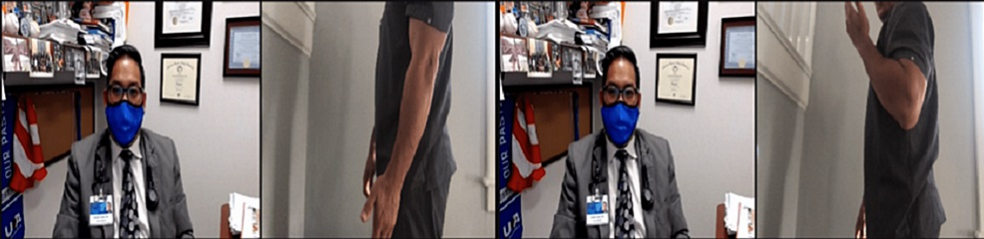
Elbow flexion range of motion testing positions. (A) Start position with the elbow at full extension. (B) End position with the elbow at full flexion.

**Figure 7:**
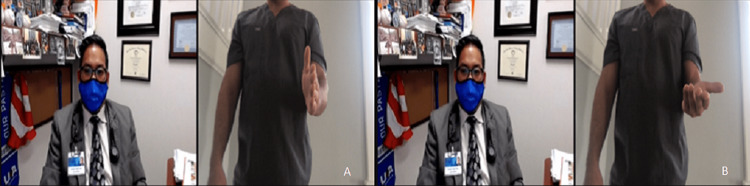
Supination range of motion testing positions. (A) Start position with the elbow flexed to 90 degrees and wrist in neutral. (B) End position with the elbow flexed to 90 degrees and palm facing the ceiling.

**Figure 8:**
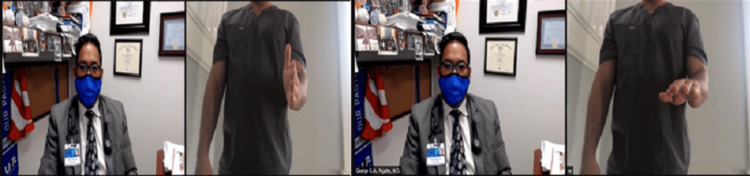
Pronation range of motion testing positions. (A) Start position with the elbow flexed to 90 degrees and wrist in neutral. (B) End position with the elbow flexed to 90 degrees and palm facing the floor.

ROM should be performed actively, which is easily observed by video. Passive ROM can be evaluated by the patient if only one elbow is affected. The unaffected arm can move the painful elbow, making sure it is not pushed beyond the point of pain. In the event the patient is accompanied by another individual, that person can be instructed on how to perform passive ROM. The passive forward flexion range is typically 130° to 150°, and the passive extension range is 0° to −10° (Figure 6). Passive supination is approximately 90°, as is passive pronation. Functional active ROM can be considered with a forward flexion range of 30° to 130°, supination of 50°, and pronation of 50° (Figures 7, 8).

**Figure 9:**
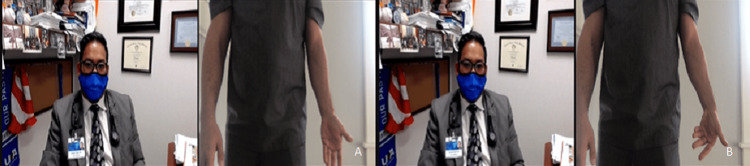
Ulnar adduction range of motion testing positions. (A) Start with the elbow in full extension and the wrist in an anatomic position. (B) End position adducts the wrist maximally.

**Figure 10:**
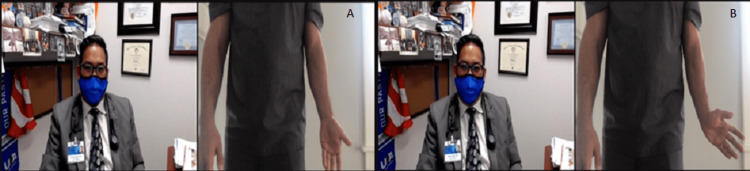
Ulnar abduction range of motion testing positions. (A) Start with the elbow in full extension and the wrist in an anatomic position. (B) End position abducts the wrist maximally.

The patient should report any popping or catching sensation in the elbow as it can be suggestive of an intra-articular body, plica or synovial fold syndrome (lateral elbow), subluxation of the ulnar nerve (medial elbow), or snapping triceps (medial elbow) (Figures 9, 10) [12].

**Figure 11:**
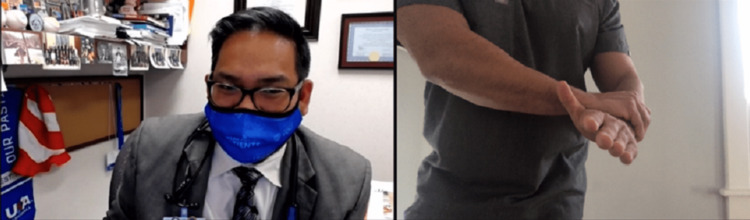
Evaluation for pronator syndrome. Instruct the patient to perform resisted pronation using the non-involved extremity. This may be indicative of pronator syndrome, as the described motions are part of the Test of Spinner, which localizes median nerve entrapment at the pronator heads. If there is only pain with wrist flexion and rotation, potential common flexor tendinosis could be present.

Strength testing can be done on video with the help of a patient companion or using weights or commonly available items, such as a can or gallon of milk, with the aim of looking for muscle weakness or to elicit pain when performed as resisted movements or weightlifting. The clinician will need to describe and demonstrate the ways resisted ROM should be tested via video. Resisted elbow extension tests the triceps and anconeus, and resisted elbow flexion tests the biceps, brachialis, and brachioradialis. Resisted wrist extension tests the common extensor tendon (CET), extensor carpi radialis longus and brevis, extensor digitorum, and extensor pollicis longus, and resisted wrist flexion tests the pronator teres, flexor carpi radialis, and flexor digitorum superficialis and profundus. Resisted supination tests the supinator and biceps, and resisted pronation tests the pronator and common flexors (Figure 11) [12].

**Figure 12:**
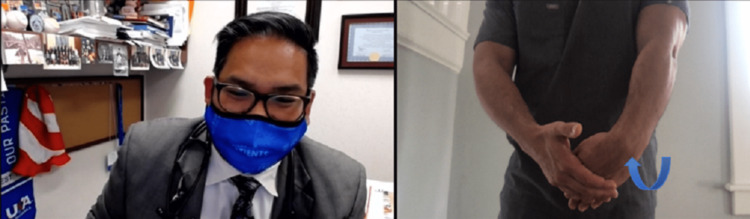
Modified Cozen test. Instruct the patient to straighten his/her elbow and maximally flex the wrist. Once in this position, have the patient apply resistance with the non-involved extremity while trying to extend the involved wrist (arrow). If pain is reproduced at the lateral epicondyle, this could support a diagnosis of lateral epicondylitis.

**Figure 13:**
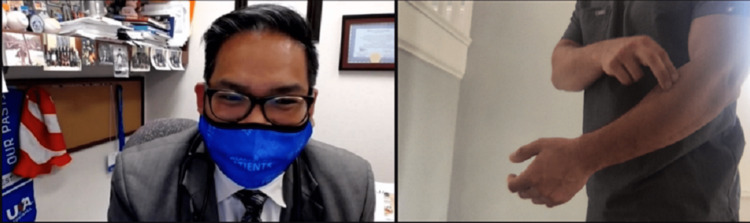
Tinel sign for radial nerve entrapment. This is Tinel’s over the radial nerve when close to the elbow crease, with the deep radial nerve and posterior interosseous nerve two to three finger breadths below the elbow crease, and the superficial radial nerve (Wartenberg syndrome) approximately halfway down the forearm. In order to assess for entrapment, instruct the patient to tap over the specified locations above.

Lateral elbow pathology is assessed by having the patient palpate over the CET. The clinician will have to demonstrate over a video where this is. Weakness or pain on finger or wrist extension (Maudsley and Cozen test) may signify pathology of the CET or posterior interosseous nerve (Figure 12) [13]. Weakness or pain with pinching an object and lifting up (chair lift and pinch test) may indicate pathology of the CET [14]. A positive Tinel sign over the supinator may indicate radial nerve pathology [15]; again, the clinician will have to demonstrate how to do the Tinel maneuver on various points of the arm (Figure 13). If there is no weakness, then likely, the pathology is prior to the arcade of Frohse or within the superficial branch of the radial nerve [16].

Radiating paresthesia with resisted supination can suggest pathology of the deep branch of the radial nerve or posterior interosseous nerve [17]. Localized pain in the lateral elbow or within the antecubital fossa may indicate distal biceps pathology or radiohumeral bursitis [18]. Sensation of instability during a pushing motion, such as a push-up, may indicate collateral ligament injury [19]; the patient can be asked to attempt a push-up on video.

Posterior elbow pathology is assessed by having the patient palpate the posterior elbow and resist elbow extension. Any pain may signify triceps or anconeus injury, and deformity with ecchymosis may indicate triceps tendon rupture or fracture [20]. A palpable nodule at the posterior elbow most often signifies an olecranon bursa [21], and care should be taken to determine if the patient has had fever, erythema, or loss of motion, which may signify an infectious etiology.

**Figure 14:**
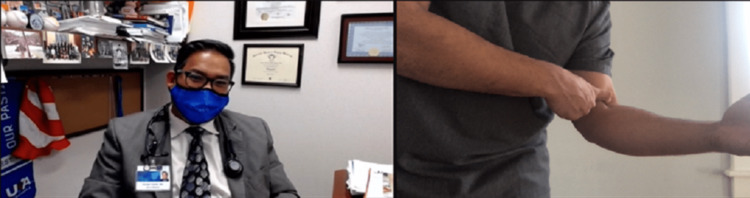
Hook test left elbow. The patient is instructed to hook his/her index finger under the common bicep tendon. If the patient is able to do this, that indicates a positive hook test, which could mean a ruptured bicep tendon is present.

Anterior elbow pathology is assessed with ROM as described earlier in addition to resisted elbow flexion, pronation, and supination. Any pain or weakness elicited may signify injury to the anterolateral elbow (i.e., biceps, brachialis, brachioradialis, supinator, CET, and radial nerve) or the anteromedial elbow (i.e., pronator/common flexor tendon and median nerve) [22]. While in 90° of elbow flexion, the patient should be able to pull gently on their distal biceps tendon as part of a hook test (Figure 14), confirming the integrity of the tendon [23]. Any loss of integrity will often be accompanied by ecchymosis and a visible mass proximal to the elbow joint [24]. Forced overpronation as part of the tilt test will elicit pain at the anterolateral elbow in the presence of distal biceps tendinopathy or radiohumeral bursitis [25]. Any ecchymosis in the antecubital fossa with paresthesia along the lateral or medial forearm may indicate a lateral or medial antebrachial nerve injury [24].

**Figure 15:**
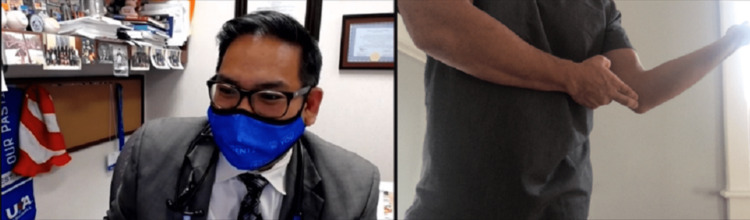
Tinel’s sign and the cubital tunnel. Instruct the patient to tap on the inside of the elbow at the medial epicondyle groove. If numbness down the forearm is elicited, this represents a positive Tinel’s sign and could signify ulnar nerve entrapment in the cubital tunnel.

**Figure 16:**
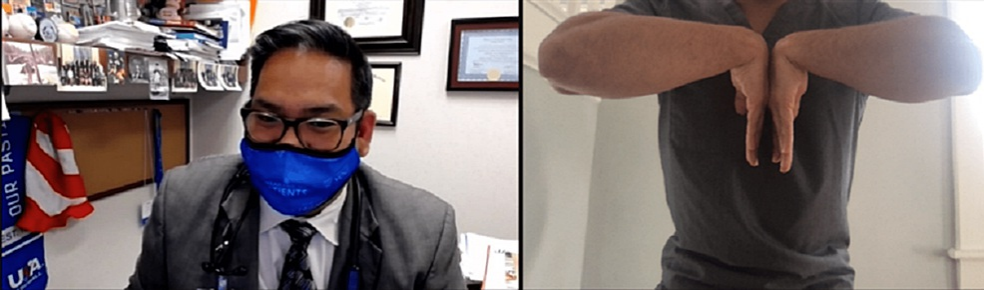
Phalen's test. Instruct the patient to hold elbows flexed with dorsal palms together and fingers pointing to the floor. If numbness is experienced, this could indicate median nerve pathology. If a snapping or popping sensation is experienced in the elbow, this could indicate ulnar nerve subluxation or pathology of the medial triceps.

**Figure 17:**
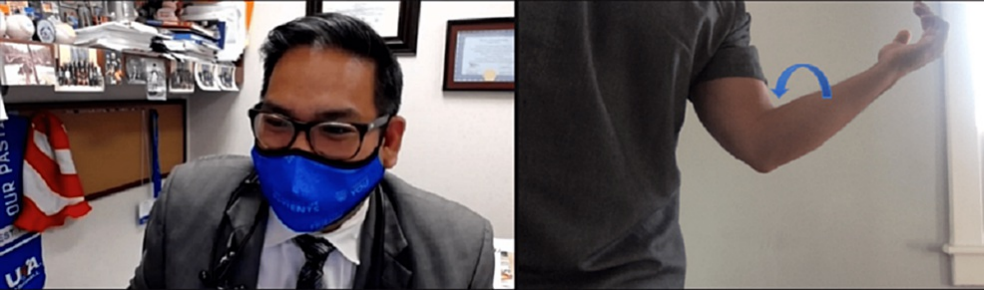
Modified milking maneuver. Instruct the patient to use the non-involved extremity to pull the involved extremity’s thumb behind the shoulder (arrow). If this maneuver elicits pain in the medial aspect of the elbow, it could indicate an injury to the UCL. UCL: ulnar collateral ligament.

Age-associated pathology must also be considered, as noted within the examination by telephone section. Medial elbow pathology is assessed by having the patient palpate over the medial side and assessing the cubital tunnel below the joint line into the medial epicondyle and epicondylar groove and extending into the medial head of the triceps. A positive Tinel sign at the cubital tunnel or medial epicondylar groove is suggestive of ulnar nerve pathology (Figure 15) [26]. Paresthesia in an ulnar distribution following elbow flexion for at least 60 seconds as part of an elbow Phalen test can further support ulnar nerve pathology [27]; the clinician can demonstrate this test (Figure 16). During flexion of the elbow, if a snapping or sliding sensation is appreciated, ulnar nerve subluxation is likely occurring [28]. If a second snapping sensation is felt during elbow flexion, this is often due to snapping triceps syndrome [29]. Valgus stress testing by a patient companion, as described in the examination by telephone section above, may elicit an ulnar collateral injury (Figure 17). This should be performed with the elbow in at least 30° of flexion to obtain the best results [30]. Resisted flexion or pronation as described above may signify pronator or common flexor tendon injury as well as median nerve entrapment from pronator syndrome [31].

## Results

A guide for questions and instructions for initial elbow self-inspection and ROM assessment is outlined in Table 1, along with common patient responses and the implications of those responses. 

**Table 1:** Medial elbow.

Medial elbow		
“Facing your palms up to the ceiling, does it hurt to press on the front of your elbow (pinkie side)?”	Affirmative response	Possible pronator tendon or common flexor tendon pathology
“With the back of your hand facing the floor, does it hurt or cause a tinging towards your pinkie when you press far inside (pinkie side) of your elbow?”	Affirmative response	Possible common flexor tendon, pronator syndrome/median nerve, ulnar collateral ligament, or ulnar nerve pathology
“Do you have weakness or pain when you flex your fingers/wrist or turn your wrist so that the palm goes from facing up to down against resistance? See Figure 8.	Affirmative response to pain or weakness	Possible pronator syndrome, as described in the motions, is part of the test of spinner localizing median nerve entrapment at the pronator heads. If there is only pain with wrist flexion and rotation, possible common flexor tendinosis
“Does tapping on the inside of the elbow at your funny bone cause you to have tingling/pain down your arm?” See Figure 9.	Affirmative response to tingling or pain	Tinel sign over the ulnar nerve at the cubital tunnel or medial epicondylar groove
“Do you have pain when you flex your elbow and face your palms up to the ceiling and pull your thumb behind your shoulder while resisting with your elbow? “Do you have pain as in the above maneuver and while trying to straighten your elbow? See Figure 10.	Affirmative response to limitation due to pain or weakness	Modified milking maneuver; positive test with pain or laxity; possible injury to the ulnar collateral ligament. A modified moving valgus stress test may be more sensitive and specific for an ulnar collateral ligament injury
“If you hold your elbows flexed so your hands are as close as possible to your shoulder for at least one minute, do you get any tingling or pain at the inside of your elbow?” See Figure 11.	Affirmative response to limitation due to tingling or pain	Phalen compression test at the elbow, possible median nerve injury or subluxation. If a popping sensation is felt, there is a possible ulnar nerve subluxation. If a second popping sensation occurs, possible pathology of the medial head of the triceps
“What tasks have you found difficult because of weakness, pain, or range of motion limitation on the elbow?”	“I am now unable to grip or twist an object because my elbow feels weak, and there is pain on the sides of my elbow.”	Possible common flexor tendinosis or medial epicondylitis. For strength testing, the clinician may also ask the patient to reproduce tasks, movements, or exercises and describe any weakness or pain felt.

As outlined above, Table 2 directs the provider-patient interaction with regard to self-inspection, palpation, and maneuvers directed at the evaluation of the medial elbow, along with perceived patient responses and their implications. 

**Table 2:** Elbow evaluation inspection and range of motion.

Physician questions	Possible patient responses	Possible implications of responses
Inspection		
“Looking at your elbows in a mirror, do you see any difference in the left elbow from the right elbow?”	“Yes, my left/right elbow doesn't fully straighten compared to the other elbow.”	Possible elbow intra-articular pathology or degenerative joint disease vs normal variant
“Do you notice any sunken, swollen, bruised, or red areas on your elbow? Has anyone else noticed such areas?”	Affirmative responses to the questions	Sunken areas could be atrophied areas; swollen, bruised, or red areas may indicate injury or infection
“Has anyone commented that your elbows look different from each other when viewed from the front, side, or back?” See Figures 1, 2.	“Yes, someone mentioned that my left/right elbow looked more prominent/swollen from the front/side/back than the other.”	Possible intra-articular swelling, triceps tendon injury, or olecranon bursal swelling/thickening
Range of motion (ROM)		
“Can you bend your elbow to touch your shoulder and then straighten it, so it is flat?” See Figure 3.	“No, my left/right elbow doesn't fully straighten compared to the other elbow.”	Possible elbow intra-articular pathology, degenerative joint disease, infection, or normal variant
“Can you reach to touch the top of your head with your arm?”	“No, my left/right side cannot touch the top of my head.”	Loss of functional ROM, implying less than 130° flexion; possible elbow intra-articular pathology, degenerative joint disease, or trauma
“Is there any difference in the amount of motion between your left/right when you bend both elbows to 90° and turn your palms to the ceiling and towards the floor?” See Figure 4.	“Yes, my left/right side does not turn as much.”	Loss of active ROM in supination and pronation; possible intra-articular or extra-articular pathology at the radioulnar or radiohumeral joint
Is there any pain when you bend both affected elbow to 90° and turn your palms to the ceiling and towards the floor?” See Figure 5.	“Yes, when I turn my palm to the ceiling/floor it produces pain.”	Pain during active ROM in supination and pronation; possible intra-articular or extra-articular pathology at the radioulnar or radiohumeral joint
“Stand with your affected elbow bent at 90° at your side with your palm up and move your wrist side to side. Does either motion produce pain?” See Figure 6.	“Yes, when I move my wrist inward or outward it produces pain.”	Pain during inward motion; possible intra-articular pathology, trauma, common flexor tender tendinitis, or somatic dysfunction at the humeroulnar joint Pain with outward motion; possible intra-articular pathology, trauma, common flexor tender tendinitis, or somatic dysfunction at the radiohumeral joint
“Stand with both of your elbows bent at 90° at your side with your palms up and move your wrists side to side. Is there a difference in the amount of motion on either side?” See Figure 7.	“Yes, the affected side moves less when I turn my wrist in/out compared to the noninjured side.”	Possible intra-articular pathology, trauma, or somatic dysfunction at the humeroulnar or radiohumeral joint

Table 3 directs the provider through questions to ask the patient for self-evaluation of the lateral elbow and outlines patient responses and their clinical implications. 

**Table 3:** Lateral elbow.

Lateral elbow		
“With the back of your hand facing towards the ceiling, does it hurt when you press on the outside of your elbow on the thumb side?” See Figure 12.	Affirmative response	Possible common extensor tendon, radial head, radial collateral ligament, and radial tunnel-nerve pathology.
“With your arm straight in front of you, flex your wrist, apply resistance to the top of your hand, and attempt to extend your wrist. Does this motion produce pain?” See Figure 13.	Affirmative response	Modified Cozen test; possible lateral epicondylitis.
“When you bring your hand all the way to your shoulder then straighten again or when turning your hand palm up to down is there pain and clicking at the thumb side of your elbow?”	Affirmative response	Possible intra-articular pathology, synovial fold syndrome/plica, or medial/lateral epicondylitis.
“With the back of your hand facing towards the ceiling, does it hurt or cause a tingling in your forearm when you press on the middle of your forearm on the thumb side?”	Affirmative response: Follow-up confirmation: “Does it cause a tingling when you try to open a closed door (resisted supination)?”	Possible radial nerve entrapment or brachioradialis pathology.
“Does tapping on the outside (thumb side) of your elbow cause you to have tingling/pain down your arm?” See Figure 14.	Affirmative response to limitation due to pain or weakness	Tinel sign over the radial nerve when close to the elbow crease, the deep radial nerve and posterior interosseous nerve two to three finger widths below the elbow crease, and the superficial radial nerve (Wartenberg syndrome) approximately halfway down the forearm.
“Do you have pain or weakness with extending out your fingers or extending your twist towards to the ceiling?”	Affirmative response to limitation due to pain or weakness	Weakness without pain is suggestive of a posterior interosseous nerve syndrome; pain and weakness may indicate common extensor tendinosis/lateral epicondylitis or radial tunnel syndrome.
“Do you have any pain at the side of your elbow or feel like your elbow will give out when doing a push-up?”	Affirmative response	Possible common extensor tendon pathology, radial collateral ligament injury, or lateral ulnar collateral ligament injury.
“What tasks have you found difficult because of weakness, pain, or range of motion limitation of the elbow?”	The responses may give clues to strength problems in the elbow; for example, “I am now unable to grip/pinch an object tightly because my elbow feels weak and there is pain on the side of my elbow.”	Depending on the description of tasks in which the patient is having weakness/pain, possible common extensor tendinosis/lateral epicondylitis or brachioradialis tendinitis/tendinosis. For strength testing, the clinician may also ask the patient to reproduce tasks/movements/exercises and describe any weakness or pain felt.

Finally, Table 4 guides the provider with instructions, patient responses, and their implications in the evaluation of the anterior and posterior elbows, along with special testing and considerations. 

**Table 4:** Anterior and posterior elbows, along with special testing and considerations.

Anterior elbow		
“Did you experience pain suddenly with weakness of elbow flexion/extension?”	Affirmative response; follow to see if any ecchymosis or visible mass	Possible distal biceps/triceps pathology
“With you elbow slightly bent can you hook your finger under your biceps tendon?” See Figure 15.	Affirmative response	Possible distal biceps tendon rupture or pathology
“Facing your palms up to the ceiling, does it hurt to press on the front of your elbow, towards the middle of your elbow, or just to the thumb side?”	Affirmative response	Possible distal biceps tendon, brachioradialis tendon, supinator tendon, or radial head pathology
“On the affected side, do you notice a defect in your biceps size or shape compared to the nonaffected side?” See Figure 16.	“Yes, my affected side has a different shape or size compared to the nonaffected side.”	Possible ruptured biceps tendon
“What tasks have you found difficult now because of weakness, pain, or range of motion limitation on the elbow?”	“I am now unable to perform elbow flexion/extension/supination/pronation because my elbow feels weak or there is pain.”	Depending on the description of tasks in which the patient is having weakness/pain, possible pathologies include biceps, pronator/supinator/brachialis tendinitis, tendinosis, or trauma.
Posterior elbow		
“With the back of your hand facing the ceiling, does it hurt on the back of the elbow?”	Affirmative response	Possible triceps tendon, olecranon bursae, or anconeus pathology
“When you press on the inside (pinkie side) of your elbow just above the elbow, do you have any pain or tingling?”	Affirmative response	Possible medial head of triceps or ulnar nerve pathology
“What tasks have you found difficult now because of weakness, pain, or range of motion limitation of the elbow?”	“I am unable to push things away from me because it hurts at the back of my elbow”	Concerning for triceps tendon injury. For strength testing, the clinician may also ask the patient to reproduce tasks, movements, or exercises and describe any weakness or pain felt.
Other important considerations		
“Did your pain begin after a fall?”	Affirmative response	Possible fracture or injury about nature of fall and any noted deformity.
“Did you recently give blood or have an intravenous catheter?”	Affirmative response	Possible lateral antebrachial nerve injury if pain is lateral; medial antebrachial nerve injury if pain is medial
“Does your elbow pain get worse at all when you look towards the ceiling and over each shoulder?” See Figure 17.	Affirmative response	Modified Spurling test; possible C5-T1 as source of radicular pain. If pain is worsened at the elbow with this maneuver, C8-T1 is highly suspicious as causative sources of pain.
If pain is lateral with loss of motion: “Are you younger, than 15? Do you throw a ball a lot? Do you feel a catching sensation?”	Affirmative responses, though the answer may vary on activity	If the patient is 7-10 years old, possible Panner disease (often including loss of motion, usually no catching) If the patient is 11-16 years old, possible capitellum osteochondritis dissecans (when a large amount of throwing, loss of motion, and catching).
If the pain is in a small child: 1.“Did an adult pull or grab the child’s hand?” 2. “Is the child holding their elbow still and refusing to move in?”	Affirmative answer(s)	Possible nursemaid’s elbow (radial head subluxation); reduction of dislocation required.

## Discussion

An evaluation over the telephone is challenging to both the patient and clinician as inherit nonverbal indications of pain and impaired function cannot be communicated in the same way. These small clues allow an experienced clinician to differentiate between common and rare pathologies while also rapidly adjusting the scope of their differential diagnosis to account for concerns beyond the chief complaint. While a history can still be elicited over the telephone, the examination relies solely on the patient's responses. Though these responses may lack the details of how a joint moved or the tactile feedback involved in a special test, the responses are still highly valuable as they often focus the clinician on the patient’s perceived functional deficit.

Working as a team with the patient, and with a bit of creativity and imagination, it is possible to conduct an examination over the telephone. Table 1 lists questions and instructions that can be given by telephone, examples of responses that patients may give, and the possible implications of these responses. The table has been further broken down into subcategories to help organize how a patient may describe their pain.

## Conclusions

Sports considerations are important for athletes experiencing elbow pain. Beyond a pandemic setting, the ability to evaluate elbow injuries across a distance can be helpful for team athletes while traveling. When imaging is needed, there are specific findings that can be described via telephone or seen on video, such as decreased ROM (elbow flexion, extension, pronation, and supination) or marked swelling, that can help clinicians know when and which tests or imaging modalities to order. Evaluation of elbow pain using telemedicine is beneficial when the patient is unable to drive or access to a local clinician is limited. Telephone and video visits allow proper triaging and may decrease unnecessary procedures, imaging, and referrals. Furthermore, evaluation, assessment, and treatment can be determined from a location that is convenient to the patient. ROM is readily visible during video visits but may be more challenging to describe during telephone visits. Whether by telephone or video, the patient’s affected elbow should always be compared with the unaffected elbow.
